# The Association of Resilience Factors with Mental Health and Substance Use outcomes Among People with and without HIV

**DOI:** 10.1093/geroni/igaf122.4381

**Published:** 2025-12-31

**Authors:** Jeremy Delgadillo, Andres Azuero, Olivio Clay, Michael Crowe, David Vance, Pariya Fazeli

**Affiliations:** The University of Alabama at Birmingham, Birmingham, Alabama, United States; The University of Alabama at Birmingham, Birmingham, Alabama, United States; The University of Alabama at Birmingham, Birmingham, Alabama, United States; The University of Alabama at Birmingham, Birmingham, Alabama, United States; The University of Alabama at Birmingham, Birmingham, Alabama, United States; The University of Alabama at Birmingham, Birmingham, Alabama, United States

## Abstract

Intrapersonal resilience factors may reduce the prevalence and severity of mental health and substance use outcomes among people living with HIV (PLWH). Cross-sectional data on intrapersonal resilience factors [i.e., resilience, self-rated successful aging (SRSA), self-perceived age], mental health [i.e., depressive symptoms, clinically relevant depressive symptoms, mental health related quality of life (MHQoL)] and substance use (i.e., illicit substance use, alcoholic drinks per week) outcomes among 174 PLWH (Mage = 51.30) and 105 people living without HIV (PLWoH; Mage = 60.10) was examined. Resilience was measured with the Connor-Davidson Resilience Scale, SRSA was a rating on a scale of 1-10, self-perceived age was subjective age minus chronological age, depression outcomes were measured with the Center of Epidemiologic Studies Depression Scale, MHRQoL was measured with the Medical Outcome Study survey, and substance use outcomes were measured with urine toxicology test and self-report. Analyses included general linear models and logistic regressions. Higher levels of resilience and SRSA were associated with fewer depressive symptoms (Resilience: B=-0.37, p < 0.01; SRSA: B=-1.24, p < 0.01), not having clinically relevant depressive symptoms (Resilience: B=-0.07, p < 0.01; SRSA: B=-.32, p < 0.01), and higher MHQoL (Resilience: B = 0.48, p < 0.01; SRSA: B = 1.10, p < 0.01), only higher resilience was associated with testing positive for THC (B = 0.06, p < 0.05). The association between SRSA and clinically relevant depressive symptoms was weaker among PLWH than PLWoH (B = 0.59, p < 0.05). Intrapersonal resilience factors may reduce the severity of depression and promote better MHQoL, and SRSA is associated with lower incidence of depression for PLWH and PLWoH alike.

